# Comprehensive study on the characterization of lysed blood samples using dual-wavelength photoacoustics

**DOI:** 10.1117/1.JBO.31.3.035001

**Published:** 2026-03-02

**Authors:** Subhadip Paul, Hari Shankar Patel, Vatsala Misra, Ravi Rani, Amaresh K. Sahoo, Ratan K. Saha

**Affiliations:** a Indian Institute of Information Technology Allahabad, Department of Applied Sciences, Prayagraj, Uttar Pradesh, India; b Raja Ramanna Centre for Advanced Technology, Laser Biomedical Applications Division, Indore, Madhya Pradesh, India; c Moti Lal Nehru Medical College, Department of Transfusion Medicine and Immunohaematology, Prayagraj, Uttar Pradesh, India

**Keywords:** photoacoustics, oxygen saturation level, hematocrit level, lysis level, Monte Carlo simulation, discrete dipole approximation

## Abstract

**Significance:**

Anemia is a global health concern, prompting the need for rapid, accurate, and noninvasive diagnostic tools. This has led to significant interest in the development of various optical tools, including photoacoustic (PA) spectroscopy for monitoring and quantification of clinically relevant blood parameters.

**Aim:**

Estimating the blood lysis level (LL) and oxygenation (${\mathrm{SO}}_{2}$) is essential for the detection of various hemolytic conditions, including anemia. The PA spectroscopy is explored here for quantifying hemolytic blood samples.

**Approach:**

*In vitro* PA measurements on human blood samples were validated through simulation studies involving discrete dipole approximation, Monte Carlo, and k-Wave methods. Blood hematocrit (H), LL, and ${\mathrm{SO}}_{2}$ levels are determined from simulated and experimental PA signals.

**Results:**

The wavelength pairs 700-905 and 700-1000 nm are found to be optimal for the estimation of H and ${\mathrm{SO}}_{2}$ with high accuracy (>90%). The correlation coefficient between the actual and evaluated LLs is calculated to be ≈0.90.

**Conclusions:**

Results show that PA measurements with a suitable combination of optical wavelengths can be used for determining the important blood parameters accurately and simultaneously. Further investigation is needed to apply the developed method under an *in vivo* setting.

## 1 Introduction

Hemolysis is the premature destruction of red blood cells (RBCs). It occurs because of the rupture of RBC membranes and causes the release of hemoglobin into the bloodstream. It is often linked with underlying health conditions.^1^ Excessive hemolysis can contribute to anemia, oxidative damage, jaundice, and renal complications, posing significant health risks.^2^^–^^5^ Anemia has always been a global issue primarily because of iron deficiency. WHO defines anemia as the hemoglobin concentration falling below 12 and 13  g/dl for females and males, respectively.^6^ In a recent study by WHO, ∼40% of all children aged 6 to 59 months, 37% of pregnant women, and 30% of women 15 to 49 years of age are affected by anemia all over the globe. In southeastern countries, particularly in the Indian subcontinent, anemia cases remain exceptionally high; more than 35% have been recorded.^7^ Various factors, including autoimmune disorders, infections, genetic abnormalities, toxins, and mechanical stress, can trigger this process.^8^^–^^11^ Early identification of hemolysis is crucial to mitigating complications, yet conventional diagnostic techniques rely on time-intensive laboratory analyses.^12^ Methods such as visual inspection, spectrometry (the most accurate one), and the heptoglobin test are commonly used for the detection and quantification of RBC lysis. Some complex and time-consuming techniques, such as lactate dehydrogenase, genetic tests, can also be performed for the same purpose.^13^

Noninvasive methods for evaluating blood oxygenation (${\mathrm{SO}}_{2}$), such as resonance Raman spectroscopy of hemoglobin and near-infrared pulse oximeters, are globally accepted alternatives, with the Masimo Pronto^®^ Pulse Co-Oximeter most prominent for its extensive clinical use and real-time *in vivo* measurement of parameters such as total hemoglobin and ${\mathrm{SO}}_{2}$.^14^^,^^15^ Blood gas analyzers remain routinely used in intensive care units and emergency departments for direct ${\mathrm{SO}}_{2}$ assessment.^16^

In recent times, the photoacoustic (PA) technique has been evolving as a promising blood characterization tool, particularly as a noninvasive tool.^17^^–^^19^ The simultaneous quantification of two important blood parameters, namely, total hemoglobin and ${\mathrm{SO}}_{2}$, can be faithfully estimated using dual-wavelength PAs. The PA signals, measured at two optical wavelengths, are utilized to obtain these parameters by exploiting spectroscopic relations.^20^^,^^21^ PA investigations on lysed blood samples have also been performed. For example, Saha et al. compared the PA signal properties of normal and lysed blood samples at 532 and 1064 nm optical wavelengths, but no quantification of blood parameters was made.^22^^,^^23^ Then, Banerjee et al. studied the dynamical variation of the PA signal profile when RBCs are undergoing lysis using a low-cost PA device employing a pulsed laser diode (PLD) source of 905 nm (spectroscopic analysis is not possible for the single-wavelength PAs).^24^ Bodera et al. examined blood clot and blood lysis *in vitro* using a high-frequency ultrasound detector-based imaging system.^25^ Note that normal and lysed blood samples are significantly different from the optical point of view, for example, (a) loss of scatterers (predominantly RBCs) with increasing lysis, (b) change of refractive index of the ambient medium reducing scattering strength of RBCs, and (c) conversion of the surrounding medium from practically nonabsorbing to considerably absorbing within the 532 to 1064 nm range. Such modifications may impact PA emission appreciably.

Besides these, many researchers all over the globe are increasingly using the PA technique for probing blood samples. For example, Yadem et al. introduced cytophone, a PA-based flow cytometer to detect malaria infection in the human body.^26^ Padmanabhan et al. developed a PA polarization–enhanced optical rotation sensing system and found chiral molecular concentration for depths up to a few millimeters.^27^ A vast amount of work has also been conducted on normal and pathological blood samples by many groups across the globe.^17^^,^^20^^,^^21^^,^^28^^–^^37^

PA emission from blood essentially depends upon its optical properties, namely, absorption coefficient ($\mu_{\mathrm{abs}}$), scattering coefficient ($\mu_{\mathrm{sca}}$), and anisotropy factor (g). Although these are bulk properties, these are governed by the microscopic features such as the spatial organization of RBCs, morphological, biophysical, and biochemical properties of individual cells.^21^^,^^33^^,^^34^ There exist a number of theoretical methods and numerical schemes, which can be used to obtain the optical absorption cross-section, scattering cross-section, and g factor of a scatterer.^38^ For instance, the Mie theory is applicable for regular shapes.^39^ The Born approximation ^40^ and Wentzel–Kramers–Brillouin approximation work well for weak scattering or slowly varying media.^41^ Other common approaches include the T-matrix method,^42^ the finite element method,^43^ the finite difference time domain method,^44^ the ray tracing method,^45^ the Monte Carlo method,^46^ etc. One popular formulation is the discrete dipole approximation (DDA).^38^^,^^47^^,^^48^ It models a scatterer having an arbitrary shape as a collection of discrete electric dipoles. The knowledge of the refractive indices of both the cell and the surrounding medium is required for its implementation. It can be used to evaluate the optical parameters of RBC as well.

Previous theoretical studies modeling PA emission from blood considered uniformly illuminated RBCs approximated as spheres and suspended in a nonabsorbing fluid medium.^20^^,^^21^^,^^33^^,^^34^ Recently, the impact of the optical parameters ($\mu_{\mathrm{abs}}$, $\mu_{\mathrm{sca}}$ and g) on quantitative PAs has been examined by us.^35^^,^^36^ Therefore, earlier works neglected the fluence variation in tissue and RBC morphology; recent studies did not consider the actual shape of RBCs and the absorbing nature of the surrounding medium (in case of lysed blood sample) while computing the optical parameters. In this study, we have addressed this gap by incorporating the exact RBC morphology for numerical calculations of optical absorption cross-section, scattering cross-section, and g factor, exploiting the DDA technique. Freely available discrete dipole scattering (DDSCAT) software implements the DDA formulation and subsequently obtained the optical properties ($\mu_{\mathrm{abs}}$, $\mu_{\mathrm{sca}}$, and g) of a blood sample at desired optical wavelengths. A series of lysed blood samples with initial hematocrit, H = 50% and oxygenation, ${\mathrm{SO}}_{2}=68\%$ have been considered. The lysis level (LL) has been varied from LL = 0 to 30%. The suspending medium has been prepared by mixing hemoglobin molecules in phosphate-buffered saline (PBS) medium or in plasma (PLS) medium. DDSCAT, Monte Carlo, and k-Wave simulations have been conducted sequentially to generate the PA signals.^36^ Analogous experimental studies (*in vitro*) have also been carried out with human blood samples. Accordingly, H, LL, and ${\mathrm{SO}}_{2}$ levels have been estimated from simulated/measured signals using dual wavelength PAs (700-905 and 700-1000 nm wavelength pairs). Simulation and experimental results reveal that accurate estimations of H are possible with accuracy >90% up to LL = 14% and for ${\mathrm{SO}}_{2}$ up to LL = 30%. The primary contributions of this work are: (i) determination of the optical parameters ($\mu_{\mathrm{abs}}$, $\mu_{\mathrm{sca}}$ and g) of lysed blood samples at some specific optical wavelengths and using the DDA model incorporating the biconcave shape of RBCs as well as optical properties of the suspending medium; (ii) generation of PA signals utilizing the k-Wave toolbox based on the fluence maps provided by the Monte Carlo simulation; (iii) measurement of PA signals from analogous human blood samples; (iv) quantification of the levels of H, LL, and ${\mathrm{SO}}_{2}$ from simulated and experimental PA signals.

## 2 Governing Theoretical Models

### 2.1 PA Wave Equation

In practice, short laser pulses illuminate a tissue sample. Photons are absorbed by the sample, causing its rapid thermo-elastic expansion, followed by emission of PA signals. The time-dependent PA wave equation in a 3D generalized coordinate system can be cast as

$$(\nabla^{2}-\frac{1}{v^{2}}\frac{\partial^{2}}{\partial t^{2}})p(r,t)=-\frac{\beta }{C_{P}}\frac{\partial H(r,t)}{\partial t}.$$ (1)

In Eq. (1), the notations v, β, and $C_{P}$ are the speed of sound in the medium, isobaric volume expansion coefficient, and isobaric specific heat of the source region, respectively; H(r,t) is the heating function, i.e., the amount of heat deposited per unit time per unit volume, and p(r,t) is the pressure at any arbitrary point at a location r at time t. The initial pressure rise in the sample due to pulsed laser heating is $p_{0}=\mu_{\mathrm{abs}}\Gamma F$ with Γ and F being the Grüneisen parameter and fluence of the laser beam, respectively. The temporal profile of a PA signal depends upon the photon weight deposition map in the tissue.

Photon propagation in a tissue can be modeled using the famous radiative transfer equation (RTE). The RTE is valid for both ballistic and diffusion regimes (<100  μm).^49^^,^^50^ An approximate version of the RTE known as the diffusion equation (DE) can also model the same faithfully when $\mu_{\mathrm{abs}}\ll \mu_{\mathrm{sca}}$. The DE is given by^51^

[ζ^.∇+(μsca+μabs)]R(r,ζ^)=μsca(∫4πR(r,ζ^)f(ζ^.ζ^′)dΩ′)+Σ(r,ζ^), (2)

where R(r,ζ^) is the time-independent radiance; ζ and $\zeta^{\prime }$ are the direction vectors of scattered and incident light; and $\Sigma (r,\hat{\zeta })$ is the source term. It may be assumed that, $H(r,t)=I_{0}(r)\mu_{\mathrm{abs}}e^{-i\omega t}$, but in general

I0(r,t)=∫4πR˜(r,ζ^,t)dΩ, (3)

and

$$F(r)=\int_{-\infty }^{\infty }I_{0}(r,t)dt,$$ (4)

where dΩ indicates the solid angle through which scattered photons pass. Note that R˜(r,ζ^,t) is the time-dependent radiance; R˜(r,ζ^,t) is related to R(r,ζ^) via the Fourier transformation operation. Equation (2) is not analytically solvable. Thus, the Monte Carlo simulation technique is generally applied. The optical properties of the tissue ($\mu_{\mathrm{abs}},\mu_{\mathrm{sca}}$ and g) have to be known for this purpose, and thus, these quantities are defined in the next sections.

### 2.2 Discrete Dipole Approximation

The complete derivation of the mathematical model of the DDA can be found else where, such as Draine et al.^47^^,^^52^^,^^53^ However, the background theory has been summarized below for the sake of completeness of this study. For a fixed target geometry in a coordinate system $\hat{x},\hat{y},\hat{z}$, we generate a dipole array. It aims to obtain a self-consistent set of dipole moments $P_{j}$, (j=1,…,N) so that $P_{j}=\alpha_{j}E_{\mathrm{loc},j}$, where $\alpha_{j}$ is the polarizability and $E_{\mathrm{loc},j}$ is the local electric field at dipole point j. As noted by Purcell and Pennypacker^54^ and Yung^55^, this can be, in terms of complex vectors, written as N simultaneous equations of the form,

$$P_{j}=\alpha_{j}(E_{\mathrm{inc},j}-\underset{m\neq j}{\sum }A_{jm}P_{m}).$$ (5)

$E_{\mathrm{inc},j}$ is the electric field at position j due to the incident plane wave, $E_{\mathrm{inc},j}=E_{0}\;\exp(ik\cdot r_{j}-i\omega t)$, and $A_{jm}P_{m}$ is the contribution to the electric field at position j due to the dipole at position m:

$$A_{jm}P_{m}=\frac{\exp(ikr_{jm})}{r_{jm}^{3}}\{k^{2}r_{jm}\times (r_{jm}\times P_{m})+\frac{\left(1-ikr_{jm}\right)}{r_{jm}^{2}}[r_{jm}^{2}P_{m}-3r_{jm}(r_{jm}\cdot P_{m})]\},\;(j\neq m).$$ (6)

where $r_{jm}=|r_{j}-r_{m}|$. Equation (6) serves to define the matrices $A_{jm}$ for j≠m. It is convenient to define also matrices $A_{jj}=\alpha_{j}^{-1}$ so that the scattering problem can be compactly formulated as a set of N inhomogeneous linear complex vector equations:

$$\overset{N}{\underset{m=1}{\sum }}A_{jm}P_{m}=E_{\mathrm{inc},j}\;(j=1,\ldots ,N),$$ (7)

here, $(A_{jm}{)}_{lk}=(A_{jm}{)}_{kl}$, i.e., the $A_{jm}$ are 3×3 symmetric matrices.

### 2.3 Extinction, Absorption, and Scattering Efficiency Factors

Once the polarization $P_{j}$ is known, the extinction coefficient factor for the grain is computed from the forward-scattering amplitude using the optical theorem^53^^,^^56^

Qext=4kaeff2|Einc|2∑j=1N Im(Einc,j*·Pj). (8)

$a_{\text{eff}}$ is the effective radius of an RBC. The absorption coefficient factor is obtained by summing over the energy dissipation rate of each dipole. By substituting $P=\alpha E_{\mathrm{inc}}$ into Eq. (8), the extinction coefficient factor can readily be obtained from the optical theorem as^47^

Qext=4kaeff2|Einc|2∑j=1NIm(P·(α−1)*P*). (9)

Note that, for isotropic polarizability, it reduces to Qext=4πk Im(α).

Thus, the absorption coefficient factor for the entire grain is^57^

Qabs=4kaeff2|Einc|2∑j=1N{Im[Pj·(αj−1)*Pj*]−2k33Pj·Pj*}. (10)

The scattering coefficient factor can, in principle, be obtained from the difference between the extinction and absorption coefficient factors:^57^

Qsca=k4πaeff2|Einc|2∫dΩ|∑j=1N[Pj−n^(n^·Pj)]exp(−ikn^·rj)|2,

where $\hat{n}$ is a unit vector in the direction of scattering. It is also of interest to evaluate the scattering asymmetry parameter, g≡⟨cos θ⟩, which is given by,^47^

g=k3πaeff2Qsca|Einc|2∫dΩ(n^·k)|∑j=1N[Pj−n^(n^·Pj)]exp(−ikn^·rj)|2.

It varies from -1 to 1.

## 3 Materials and Methods

### 3.1 Numerical Investigation

#### 3.1.1 Calculation of optical absorption coefficient and complex refractive index

A healthy RBC typically occupies a volume of $V_{\mathrm{RBC}}=91.52\;\mathrm{fL}$ and retains 281 million hemoglobin molecules having a molar mass of 64500 g; 66% of $V_{\mathrm{RBC}}$ is water.^36^^,^^58^ Based on these, the optical absorption coefficient for the semi-solid medium present inside RBC could be expressed as

$$\mu_{\mathrm{abs}}^{\mathrm{RBC}}=2.303(C_{\mathrm{HbO}}\varepsilon_{\mathrm{HbO}}+C_{\mathrm{Hb}}\varepsilon_{\mathrm{Hb}})+0.66\mu_{\mathrm{abs}}^{W},$$ (11)

where $C_{\mathrm{HbO}}$ and $\varepsilon_{\mathrm{HbO}}$ are the molar concentration and extinction coefficient for oxy-hemoglobin (HbO), respectively; the same quantities for deoxy-hemoglobin (Hb) are denoted by $C_{\mathrm{Hb}}$ and $\varepsilon_{\mathrm{Hb}}$, respectively; $\mu_{\mathrm{abs}}^{W}$ is the optical absorption coefficient for water.^59^ Accordingly, real and imaginary parts of the refractive index for the same cellular matrix could be estimated to be,^60^

$${\text{n}}_{r}^{\mathrm{RBC}}={\text{n}}_{r}^{M}[B(C_{\mathrm{HbO}}+C_{\mathrm{Hb}})+1],$$ (12)

$$n_{i}^{\mathrm{RBC}}=\frac{\mu_{\mathrm{abs}}^{\mathrm{RBC}}\lambda }{4\pi },$$ (13)

respectively, ${\text{n}}_{r}^{M}$ is the refractive index of the PBS/PLS medium. Here, B is the wavelength-dependent specific refractive index increment.^61^^,^^62^ The numerical values of these quantities for an RBC with oxygen saturation ${\mathrm{SO}}_{2}=0.68$ could be computed to be, $\mu_{\mathrm{abs}}^{\text{RBC}}=9.070$, 12.563 and $9.258\;{\mathrm{cm}}^{-1}$, $n_{r}^{\text{RBC}}=1.418$, 1.415 and 1.417, and $n_{i}^{\text{RBC}}=5.050\times 10^{-5}$, $9.050\times 10^{-5}$ and $7.370\times 10^{-5}$ at 700, 905, and 1000 nm, respectively, assuming $C_{\mathrm{HbO}}+C_{\mathrm{Hb}}=5.1\times 10^{-3}\;\text{mole}/L$.

It may be noted that the absorption contrast between Hb and HbO is large at wavelengths around 700 nm.^59^ This wavelength thus becomes very sensitive to changes in blood ${\mathrm{SO}}_{2}$. The 1000 nm wavelength lies in the near-infrared window, where optical penetration depth is higher, light scattering is reduced, and also, the absorption contrast between Hb and HbO is very strong. These two wavelengths are situated on either side of the isosbestic point (800 nm). In addition, absorption contrast is opposite at 700 and 1000 nm, further enhancing the accuracy of dual-wavelength ${\mathrm{SO}}_{2}$ estimation. The 905 nm wavelength was selected to compare experimental data provided by bulky (Nd:YAG) and portable (PLD) lasers (described later). For the above reasons, these wavelengths were chosen in this study.

The optical absorption coefficient for the extracellular medium significantly changes in the presence of freely suspending hemoglobin molecules, occurring because of the lysis of RBCs. It could be estimated by evaluating the following equation:

$$\mu_{\mathrm{abs}}^{\mathrm{LM}}=\frac{1}{\left(1-H+H\times \mathrm{LL}\right)}[H\times \mathrm{LL}\mu_{\mathrm{abs}}^{\mathrm{RBC}}+(1-H)\mu_{\mathrm{abs}}^{M}],$$ (14)

where LL is the lysis level and H is the hematocrit (at LL=0%) of the sample; the subscript LM indicates PBS or PLS based lysed medium. Table 1 displays the computed values of $\mu_{\mathrm{abs}}^{\mathrm{LM}}$ and refractive index for a series of blood samples having different lysis levels but with H=0.50 and ${\mathrm{SO}}_{2}=0.68$. The same quantity for the whole blood ($\mu_{\mathrm{abs}}^{\mathrm{BS}}$) could also be determined as (by adding the contributions from intact RBCs and surrounding medium),

$$\mu_{\mathrm{abs}}^{\mathrm{BS}}=H(1-\mathrm{LL})\mu_{\mathrm{abs}}^{\mathrm{RBC}}+(1-H+H\times \mathrm{LL})\mu_{\mathrm{abs}}^{\mathrm{LM}},$$ (15)

subscript BS states blood sample; its numerical values are included in the same table (columns 7 and 11 for PBS and PLS media, respectively, of Table 1).

**Table 1:** Tabulated values of the real (${\text{n}}_{r}^{\mathrm{LM}}$) and imaginary (${\text{n}}_{i}^{\mathrm{LM}}$) components of the refractive index for the suspending lysed media (PBS/PLS based) across different stages of lysis.

λ (nm)	Blood sample		PBS				PLS			
	H (%)	LL (%)	$\mu_{\mathrm{abs}}^{\mathrm{LM}}$ (${\mathrm{cm}}^{-1}$)	$n_{r}^{\mathrm{LM}}$	$n_{i}^{\mathrm{LM}}$ ($\times 10^{-5}$)	$\mu_{\mathrm{abs}}^{\mathrm{BS}}$ (${\mathrm{cm}}^{-1}$)	$\mu_{\mathrm{abs}}^{\mathrm{LM}}$ (${\mathrm{cm}}^{-1}$)	$n_{r}^{\mathrm{LM}}$	$n_{i}^{\mathrm{LM}}$ ($\times 10^{-5}$)	$\mu_{\mathrm{abs}}^{\mathrm{BS}}$ (${\mathrm{cm}}^{-1}$)
700	50	0	0.021	1.331	0.010	4.545	0.042	1.344	0.020	4.556
	47	6	0.533	1.336	0.300	4.545	0.553	1.349	0.310	4.556
	43	14	1.132	1.342	0.630	4.545	1.151	1.354	0.640	4.556
	40	20	1.529	1.346	0.850	4.545	1.547	1.358	0.860	4.556
	35	30	2.109	1.351	1.170	4.545	2.125	1.364	1.180	4.556
905	50	0	0.053	1.328	0.040	6.307	0.126	1.338	0.090	6.344
	47	6	0.761	1.333	0.550	6.307	0.830	1.343	0.600	6.344
	43	14	1.589	1.339	1.140	6.307	1.654	1.349	1.190	6.344
	40	20	2.138	1.343	1.540	6.307	2.199	1.353	1.580	6.344
	35	30	2.940	1.348	2.120	6.307	2.996	1.358	2.160	6.344
1000	50	0	0.464	1.327	0.370	4.861	0.305	1.337	0.240	4.781
	47	6	0.962	1.332	0.770	4.861	0.812	1.342	0.650	4.781
	43	14	1.544	1.338	1.230	4.861	1.405	1.348	1.120	4.781
	40	20	1.930	1.342	1.540	4.861	1.797	1.352	1.430	4.781
	35	30	2.494	1.348	1.980	4.861	2.371	1.358	1.890	4.781

#### 3.1.2 DDA simulation

**Fig. 1:**
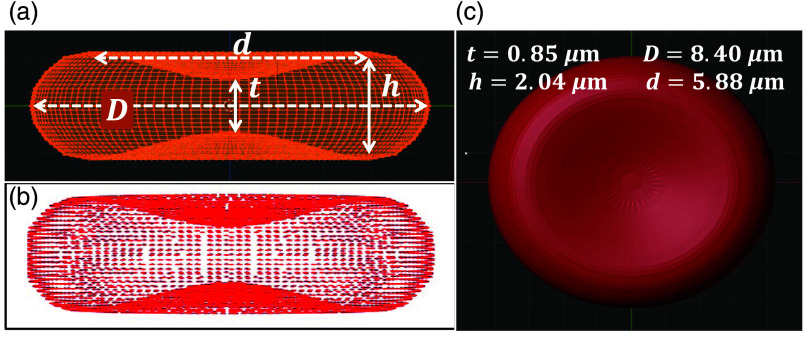
Visualization of red blood cell (RBC) geometry. (a) Side view of the RBC structure. (b) Dipole distribution used for electromagnetic modeling. (c) Top view highlighting the characteristic biconcave shape.

The RBC model was designed in Blender 4.2, an open-source software. Normal RBC looks like a biconcave disk.^58^^,^^63^^,^^64^ Figure 1 shows the meridional cross-section of a biconcave RBC, defined by four critical morphological parameters: the diameter (D), the dimple thickness (t), the maximum thickness (h), and the diameter (d) of the circle determining the location of the maximum thickness. The chosen values of that morphological parameter were D=8.40  μm, t=0.85  μm, h=2.04  μm, and d=5.88  μm providing an effective radius, $a_{\mathrm{eff}}=2.79\;\mu m$.

The DDSCAT module is an open-source Fortran-based algorithm.^57^ The DDSCAT version 7.3.1 was employed, and single precision mode was used to execute the DDA simulations. The DDSCAT simulation was configured by selecting specific options for key parameters. Radiative torque calculations were disabled. The conjugate gradient solver was chosen as preconditioned Bi-Conjugate Gradient Stabilized. The fast Fourier transforms were performed using the generalized prime factor algorithm method. The dipole polarizability was computed using the generalized kernel dipole layered dipole regularization technique. Finally, binary output files were avoided during the simulation. The computational volume in DDSCAT was set to 230×230×80  voxels containing cubic lattice points. The RBC model was discretized into $∼1.6\times 10^{6}$ dipoles for the wavelengths of interest (i.e., 700, 905, 1000 nm) using the DDSCAT-convert module available on nanoHUB.org [see Fig. 1(b)]. The refractive indices ($n_{r}$ and $n_{i}$) of the RBC and the surrounding medium were determined and presented in Table 1. The error tolerance and maximum number of iterations were set to $10^{-5}$ and 1000, respectively. A total of 125 orientation-average was considered to yield $Q_{\mathrm{abs}},Q_{\mathrm{sca}}$ and g. All computations were performed on a high-performance workstation equipped with 256 GB RAM, an AMD Ryzen Threadripper PRO 5965WX 24-core processor (3.80 GHz), 932 GB SSD, and 7.28 TB HDD storage. Depending on the chosen H and LL levels, $\mu_{\mathrm{abs}}^{\mathrm{BS}}$ and $\mu_{\mathrm{sca}}^{\mathrm{BS}}$ were calculated using the equations given below:^35^^,^^36^^,^^65^

$$\mu_{\mathrm{abs}}^{\mathrm{BS}}={\frac{H}{V}}_{\mathrm{RBC}}Q_{\mathrm{abs}}\pi a_{\mathrm{eff}}^{2},$$ (16)

$$\mu_{\mathrm{sca}}^{\mathrm{BS}}={\frac{H}{V}}_{\mathrm{RBC}}(1-H{)}^{2}Q_{\mathrm{sca}}\pi a_{\mathrm{eff}}^{2},$$ (17)

numerical value of g was provided by the DDSCAT as well.

#### 3.1.3 Monte Carlo and k-Wave simulations

The Monte Carlo multilayered (MCML) algorithm was utilized to model photon transport in homogeneous blood samples vis-á-vis to obtain the fluence matrix (F).^46^ The simulation domain consisted of a cubic tissue volume with dimensions 200×200×200  voxels, where each voxel was fixed to be $dx\times dy\times dz=0.005\times 0.005\times 0.005\;{\mathrm{cm}}^{3}$. The optical properties ($\mu_{\mathrm{abs}}^{\mathrm{BS}}$, $\mu_{\mathrm{sca}}^{\mathrm{BS}}$, and g) at a particular wavelength of each voxel were assigned according to Table 2, corresponding to the distinct blood sample under investigation. We introduced ±1% random fluctuations in $\mu_{\mathrm{abs}}^{\mathrm{BS}}$ for 10% of randomly selected voxels to introduce a little randomness, simulating a real condition. The incident laser beam had a diameter of $D_{B}=0.10\;\mathrm{cm}$ and featured a uniform lateral spatial profile along with a delta-function temporal profile. For each simulation, $2\times 10^{6}$ photons were launched with reflections enabled only at the top surface (z=0), whereas for the side and bottom walls, reflections were disabled. The fluence maps were generated for a specific sample at λ=700, 905, and 1000 nm. A schematic of the simulation setup was shown in Figure 3 of Ref. 35.

**Table 2:** DDSCAT-based computation of absorption, scattering, and anisotropy coefficients across different lysis levels in an ambient environment (initial surrounding medium was PBS or PLS).

H = 50% ${\mathrm{SO}}_{2}=68\%$		DDSCAT (PBS)					DDSCAT (PLS)				
λ (nm)	LL (%)	$Q_{\mathrm{abs}}$ ($\times 10^{-5}$)	$\mu_{\mathrm{abs}}^{\mathrm{BS}}$ (${\mathrm{cm}}^{-1}$)	$Q_{\mathrm{sca}}$	$\mu_{\mathrm{sca}}^{\mathrm{BS}}$ (${\mathrm{cm}}^{-1}$)	g	$Q_{\mathrm{abs}}$ ($\times 10^{-5}$)	$\mu_{\mathrm{abs}}^{\mathrm{BS}}$ (${\mathrm{cm}}^{-1}$)	$Q_{\mathrm{sca}}$	$\mu_{\mathrm{sca}}^{\mathrm{BS}}$ (${\mathrm{cm}}^{-1}$)	g
700	0	389.4	5.203	3.045	1017.2	0.991	385.0	5.144	2.738	914.5	0.992
	6	387.8	4.870	2.927	1032.8	0.991	383.0	4.810	2.604	918.6	0.993
	14	385.7	4.432	2.785	1040.9	0.992	380.4	4.371	2.424	905.1	0.993
	20	384.2	4.107	2.689	1034.9	0.992	378.6	4.047	2.287	880.2	0.994
	30	381.9	3.572	2.530	999.8	0.993	375.7	3.514	2.065	816.3	0.994
905	0	539.9	7.214	2.571	858.9	0.989	534.6	7.143	2.317	773.9	0.990
	6	537.4	6.750	2.453	865.6	0.990	531.7	6.678	2.166	764.3	0.991
	14	534.2	6.138	2.297	857.5	0.990	528.1	6.068	1.969	735.3	0.991
	20	532.0	5.687	2.182	839.8	0.990	525.5	5.617	1.830	704.2	0.991
	30	528.5	4.943	1.994	788.1	0.991	521.2	4.875	1.609	635.8	0.992
1000	0	399.4	5.337	2.461	822.0	0.988	395.5	5.285	2.206	737.0	0.989
	6	397.5	4.992	2.335	823.9	0.988	393.3	4.940	2.050	723.3	0.989
	14	395.0	4.539	2.170	810.0	0.989	390.5	4.487	1.850	690.5	0.990
	20	393.3	4.204	2.051	789.4	0.989	388.6	4.154	1.711	658.6	0.990
	30	390.6	3.654	1.859	734.6	0.990	385.5	3.606	1.498	591.9	0.990

**Fig. 2:**
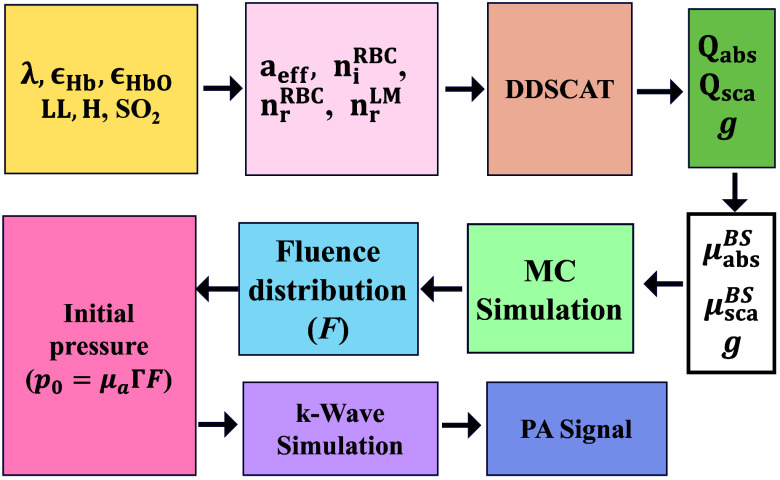
Workflow diagram illustrating the simulation pipeline implemented for modeling various lysis levels of RBC samples in PBS and PLS suspending media.

After calculating the fluence matrix (F), the k-Wave toolbox was used to perform 3D acoustic simulations (Fig. 2). The initial pressure rise was computed as $p_{0}=\Gamma \mu_{\mathrm{abs}}F$, where Γ=1 for all samples. The simulation domain size was 240×240×280 with a grid spacing of dx=dy=dz=0.005  cm. A perfectly matched layer (PML) of thickness 0.10 cm was employed, with an anisotropic absorption coefficient of 100  np/m to diminish boundary reflections. A spherically focused circular aperture ultrasound transducer (UST) with a radius of 0.05 cm was placed at (120, 120, 260) grid location. The focal point of the transducer was fixed at the center of the computational domain (120, 120, 100). The sensor consisted of 317 grid points, with a center frequency of 5 MHz and a 70% fractional bandwidth. The speed of sound ($v_{s}=v_{M}=1500\;m/s$) and density ($\rho_{s}=\rho_{M}=1000\;\mathrm{kg}/m^{3}$) remained uniform throughout the computational domain. The Courant–Friedrichs–Lewy number was set to 0.3, achieving numerical stability and providing a time step of 10 ns. A Gaussian noise was added to the simulated pressure signals to achieve a 40 dB signal-to-noise ratio.

### 3.2 Experimental Procedure

#### 3.2.1 Sample preparation

Fresh human whole blood samples were obtained from a local blood bank. All five donors were healthy individuals aged between 25 and 40 years. The samples were collected in vacutainer tubes containing EDTA as an anticoagulant agent. A complete report of various blood parameters for each donor is provided in Table 3. This project was cleared a priori by the institute’s ethics committee (File no. IEC02/2020).

**Table 3:** Summary of demographic details and hematological indices of blood donors, showing inter-individual variability in key parameters including hematocrit (H), total hemoglobin (THB), mean corpuscular volume (MCV), mean corpuscular hemoglobin (MCH), mean corpuscular hemoglobin concentration (MCHC), red cell distribution width (RDW), and red blood cell count (RBC).

Donor	Age (year)	Gender	H (%)	THB (g/dL)	MCV (fL)	MCH (pg)	MCHC (g/dL)	RDW (%)	RBC ($10^{6}/\mu L$)
1	28	Male	44.3	14.7	78.7	26.2	33.2	14.7	5.63
2	32	Female	41.8	14.0	81.2	27.2	33.4	13.7	5.13
3	35	Male	49.1	16.4	89.8	29.9	33.3	15.0	5.46
4	40	Male	54.8	16.4	77.7	23.3	30.0	19.0	7.05
5	35	Male	42.6	13.9	99.6	32.4	32.5	13.3	4.28

**Fig. 3:**
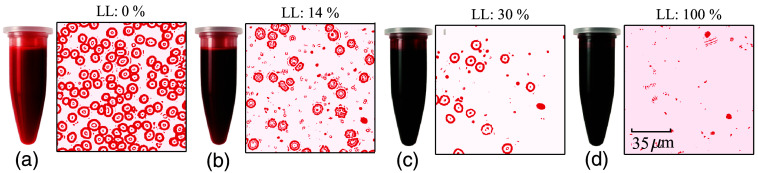
(a)–(d) Blood samples at the lysis levels of LL = 0%, 14%, 30%, and 100%, with corresponding microscopic images of smeared samples on glass slides. Scale bar: 35  μm (edited with Pseudo color).

For sample preparation, the blood samples were centrifuged at 1000×g for 15 min at room temperature. Blood plasma was carefully extracted, and the buffy coat layer was also meticulously discarded. The packed RBCs were then evenly divided into two separate tubes. Plasma was poured into one tube, whereas phosphate-buffered saline (PBS) was added to the other, ensuring that both samples maintained the same hematocrit level of 50%. This procedure was consistently followed for all donors to prepare a total of 10 samples. In the next step, individual samples were further divided into two parts. One part was subject to complete lysis using an ultrasound sonicator (Qsonica, XL-2000 series, 5W emission, 5-second pulse). The lysed solution was further centrifuged to discard the debris. The other half was left intact. A series of lysed samples was prepared by mixing the 100% and 0% lysed solutions in different ratios, achieving LL=0%, 6%, 14%, 20%, and 30% lysis levels. A blood sample of ∼800  μL was placed in a cylindrical sample holder for a subsequent PA experiment. Some representative images of lysed blood samples and the corresponding microscopic images are demonstrated in Fig. 3. The number of cells decreases with increasing lysis.

#### 3.2.2 PA signal detection

**Fig. 4:**
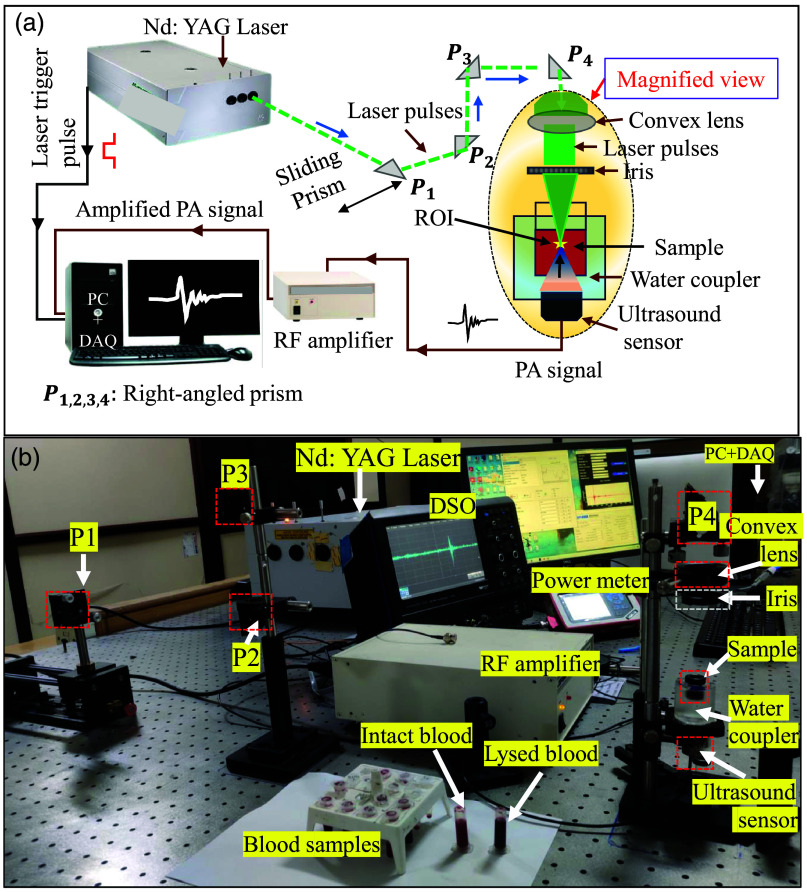
(a) Schematic diagram and (b) photograph of the Nd:YAG laser-based PA experimental setup.

The PA experimental setup is shown in Fig. 4. A Nd:YAG laser source (EKSPLA NT352, Vilniaus, Lithuania, optical parametric oscillator–enabled) was employed to perform the measurements. It is a solid-state, Q-switched, tunable, and pulsed laser source emitting pulses of 6 ns width at a 10 Hz repetition frequency. This instrument is capable of emitting a range of wavelengths from 335 to 2500 nm (excluding the optical range 501 to 659 nm, but including 532 nm). Four prisms $\left(P_{1},P_{2},P_{3},P_{4}\right)$ were used to guide the laser beam. The beam was shaped and focused to a spot using an iris and a convex lens. A spherically focused ultrasound transducer (ISR053) with a center frequency of 5 MHz, bandwidth of 70%, and focal length of 25 mm was mounted at the bottom of the sample holder. It was acoustically coupled through water, as shown in Fig. 4(a). The changes in PA signals could be captured well using this transducer. A data acquisition card (ADLINK PCIe-9852) was used to record the captured PA signals at a sampling frequency of 50 MHz; each signal contained 5000 samples. For each blood sample, a total of 100 RF signals were stored. The PA measurements were conducted at optical wavelengths of 700, 905, and 1000 nm, using optical fluences of 5.5, 19.0, and $47\;\mathrm{mJ}/{\mathrm{cm}}^{2}$, respectively, to remain within the ANSI safety limits.^66^ The optical fluence at the plane of illumination (sample) at each wavelength was measured by a power meter (Thorlabs, PM100D, Newton, New Jersey, United States). The individual PA signals were normalized by the laser fluence and also adjusted to a specific gain of 50 dB (arbitrarily chosen). This step was carried out for all PA signals before quantitative analysis. The distinguishability of PA signals associated with various lysis levels was examined via a paired t-test for all donors and at every wavelength. The exact H level was determined using a micro-capillary centrifuge rotating at 8500 rpm for 2 min. UV–Vis spectrophotometric data were also collected for the blood samples with 0% and 30% lysis levels between the wavelength range 500 to 1100 nm (PerkinElmer Lambda 365+, Waltham, Massachusetts, United States). The blood ${\mathrm{SO}}_{2}$ level was assessed utilizing measured UV–Vis data.

In addition, a customized PLD setup was used to study the dynamic aspects of the lysis process. The setup was similar to that of Fig. 4. Instead of the Nd:YAG laser, a laser beam from a PLD source (Laser Components, 905D583J08S, Olching, Germany) emitting 905 nm wavelength irradiated the blood sample under investigation; it was connected to a driver (LDP-V 240-100 V3.3) and a power supply (LDP-V KIT). A combination of collimating and aspheric lenses was used for beam shaping. The pulse width was ∼120  ns, and the pulse repetition rate was set to 100 Hz.

### 3.3 Analysis of PA Signals and Quantification of the Hematocrit, Blood Oxygenation, and Lysis Levels

The total hemoglobin concentration (THB) and oxygen saturation of a blood sample can be assessed if PA signals are measured at two optical wavelengths by exploiting the following equation:^20^^,^^21^

$$\mathrm{THB}=\frac{P_{p}(\lambda_{1})\Delta \epsilon^{\left(\lambda_{2}\right)}-P_{p}(\lambda_{2})\Delta \epsilon^{\left(\lambda_{1}\right)}}{\epsilon_{\mathrm{Hb}}^{\left(\lambda_{1}\right)}\epsilon_{\mathrm{HbO}}^{\left(\lambda_{2}\right)}-\epsilon_{\mathrm{Hb}}^{\left(\lambda_{2}\right)}\epsilon_{\mathrm{HbO}}^{\left(\lambda_{1}\right)}}$$ (18)

and

$${\mathrm{SO}}_{2}=\frac{P_{p}(\lambda_{2})\epsilon_{\mathrm{Hb}}^{\left(\lambda_{1}\right)}-P_{p}(\lambda_{1})\epsilon_{\mathrm{Hb}}^{\left(\lambda_{2}\right)}}{P_{p}(\lambda_{1})\Delta \epsilon_{b}^{\left(\lambda_{2}\right)}-P_{p}(\lambda_{2})\Delta \epsilon_{b}^{\left(\lambda_{1}\right)}},$$ (19)

respectively and $\Delta \epsilon_{b}^{\left(\lambda \right)}=\epsilon_{\mathrm{HbO}}^{\left(\lambda \right)}-\epsilon_{\mathrm{Hb}}^{\left(\lambda \right)}$. Here, $P_{p}$ is the peak-to-peak amplitude of the PA signal. The blood sample with the highest hematocrit for each batch was considered the calibration sample, using which the H levels were determined for other samples. The LL was calculated to be

$$\mathrm{LL}=\frac{\left|H_{\text{initial}}-H_{\text{current}}\right|}{H_{\text{initial}}}\times 100\%,$$ (20)

where $H_{\text{initial}}$ is the initial H (known a priori) and the same quantity is designated as $H_{\text{current}}$ for a lysed sample under study.

## 4 Results

### 4.1 Simulation Results

**Fig. 5:**
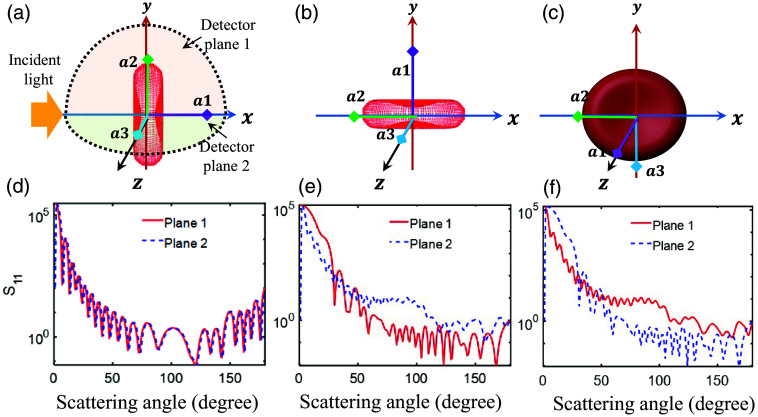
(a)–(c) The schematic diagram showing various orientations of RBC; a1, a2, and a3 are the axes associated with the object frame. The direction of the incident light and detection planes are defined with respect to the laboratory frame in panel (a). (d)–(f) The corresponding plots of the angular distribution of scattering amplitude ($S_{11}$) computed at two specific scattering planes for 1000 nm incident optical wavelength.

**Fig. 6:**
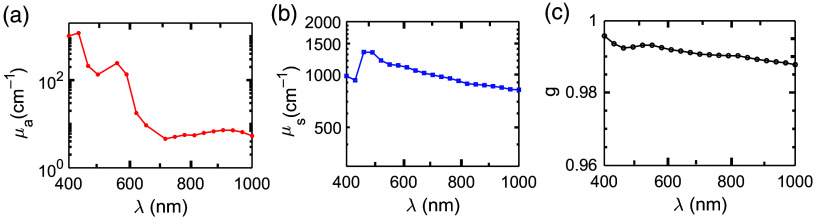
Plots of (a) absorption coefficient ($\mu_{\mathrm{abs}}$), (b) scattering coefficient ($\mu_{\mathrm{sca}}$), and (c) anisotropy coefficient (g) spectra for a blood sample with hematocrit H = 50%, ${\mathrm{SO}}_{2}=68\%$, and LL=0%. The results are generated using the DDSCAT module with PBS as the surrounding medium.

The angular scattering patterns for a healthy RBC generated by the DDSCAT module are shown in Fig. 5. We considered three orientations of the scattering object as can be visualized from Figs. 5(a)–5(c). The direction of the incident light is shown in Fig. 5(a). The scattering planes are chosen perpendicular to each other, i.e., lying on the xy and zx-planes, and the detectors are placed over 0 to 180 deg. Figures 5(d)–5(f) plot the corresponding angular distributions of scattering amplitude ($S_{11}$) for 1000 nm incident light recorded at those scattering planes. It is evident from Fig. 5(d) that the calculated $S_{11}$ for both the detection planes overlap because the source looks identical for these planes. Similarly, Figs. 5(e) and 5(f) present that $S_{11}$ curves are getting separated and switch position because of the symmetry possessed by the biconcave shape. The $\mu_{\mathrm{abs}}$, $\mu_{\mathrm{sca}}$, and g spectra calculated using the DDSCAT software are plotted in Figs. 6(a)–6(c) for an optical spectral range of 400 to 1000 nm with H = 50%, ${\mathrm{SO}}_{2}=68\%$, and LL = 0% and for the PBS suspending medium.

**Fig. 7:**
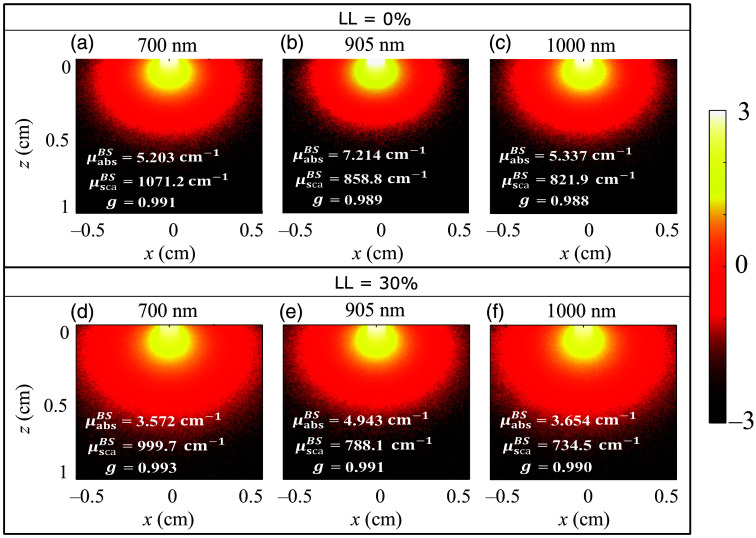
Cross-sectional views of fluence distribution in PBS-based lysed blood samples at 700, 905, and 1000 nm for LL = 0% and 30% lysis levels with ${\mathrm{SO}}_{2}=68\%$ and initial hematocrit level, H = 50%.

Photon-weight deposition maps generated by the MC simulation are shown in Fig. 7 for blood samples with an initial hematocrit level, H = 50% and lysis levels LL = 0% and 30% for the illuminating wavelengths of 700, 905, and 1000 nm. The nominal oxygenation level is fixed at ${\mathrm{SO}}_{2}=68\%$, and PBS is the suspending medium. A color bar tied to the figure quantifies the color code. The optical parameters ($\mu_{\mathrm{abs}}^{\mathrm{BS}}$, $\mu_{\mathrm{sca}}^{\mathrm{BS}}$, and g) are provided in each figure. The photons incident from the top surface, z=0. It can be seen from this figure that photons are diffused less and vis-á-vis penetration is reduced in the blood sample with LL = 0% compared with that of LL = 30% [compare top and bottom rows of Fig. 7]. This is because $\mu_{\mathrm{abs}}^{\mathrm{BS}}$ is higher at the former sample than the latter one and is also true for all incident optical wavelengths.

**Fig. 8:**
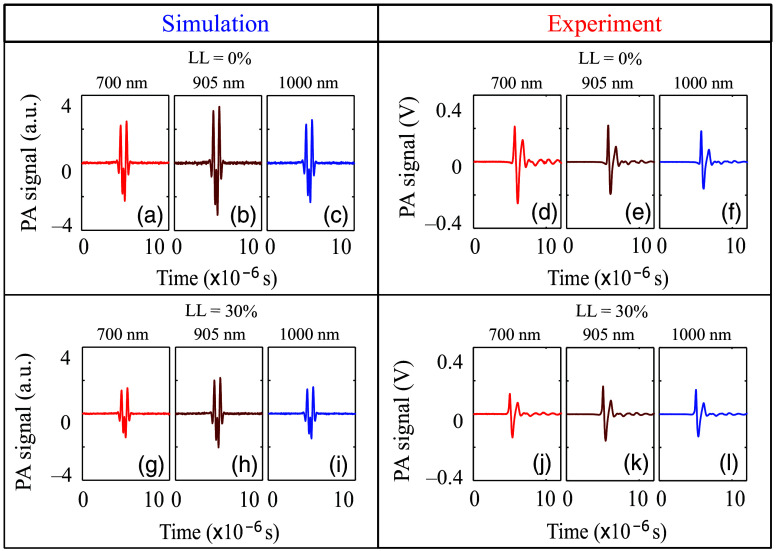
Left block: demonstration of the simulated PA signals for LL = 0% [(a)–(c), top panel] and LL = 30% [(g)–(i), bottom panel] lysed blood samples at 700, 905, and 1000 nm wavelengths, ${\mathrm{SO}}_{2}$ = 68% and initial hematocrit level, H = 50%. Right block: Similar plots for the experimentally measured PA signals for LL = 0% [(d)–(f), top panel] and LL = 30% [(j)–(l), bottom panel) for the sample collected from donor 1.

The corresponding PA signal profiles computed using the k-Wave toolbox for LL = 0% and 30% are shown in the left panel of Figs. 8(a)–8(c) and 8(g)–8(i), respectively. The PA signal strength at LL = 0% is greater than that of LL = 30%. Moreover, signal amplitude is consistently stronger at 905 nm than at the other wavelengths. This is attributed to the higher values of $\mu_{\mathrm{abs}}^{\mathrm{BS}}$ at 905 nm for both LL = 0% and 30% than those of the other wavelengths, as expected.

**Fig. 9:**
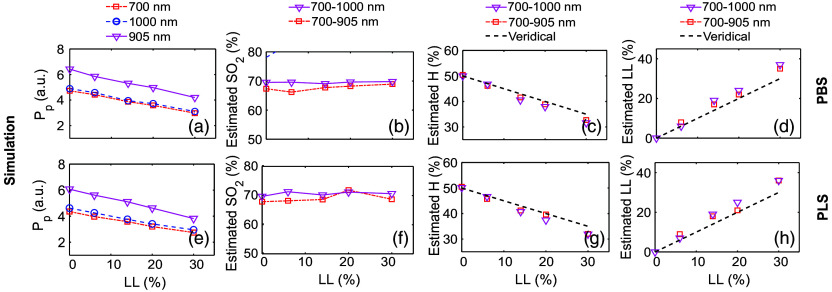
Analysis of simulated PA signals- amplitude variation (a) and (e) with LL; plots of estimated blood oxygenation (b), (f), hematocrit (c), (g) and lysis level (d), (h) for both PBS and PLS media.

Figures 9(a) and 9(e) display how $P_{p}$ varies with lysis level ranging from 0% to 30% for PBS and PLS suspending media, respectively, at the optical wavelengths of 700, 905, and 1000 nm. The $P_{p}$ exhibits an almost linear decrease with increasing LL. In both media, $P_{p}$ values at 905 nm are higher than those at 700 and 1000 nm. Further, the data points for 700 and 1000 nm are nearly overlapping. This observation is consistent with the fluence distribution maps (Fig. 7) and the PA signal profiles [see Figs. 8(a)–8(c) and 8(g)–8(i), left block]. It is clear that PA amplitude drops by nearly 35% at 700 and 905 nm as the LL increases from 0 to 30%, and the same value is ∼39% at 1000 nm [see Fig. 9(a)].

The estimated ${\mathrm{SO}}_{2}$ values for PBS and PLS media are plotted in Figs. 9(b) and 9(f), respectively, alongside the corresponding nominal LL values across the same wavelength combinations. For the 700-905 and 700-1000 nm wavelength pairs, the ${\mathrm{SO}}_{2}$ estimates remain nearly constant with increasing LL. The maximum estimation errors in PBS are ∼1.6% and 3.6% for these pairs, respectively. In PLS medium, the corresponding errors are 2.6% and 3.9%, respectively. These values are tabulated in Tables 5 and 6, columns 4, 5 and 7, 8; rows 3 to 7. The ${\mathrm{SO}}_{2}$ levels assessed using the 905-1000 nm combination have demonstrated significant fluctuations (data not shown). Logically, this combination is not suitable for the estimation of ${\mathrm{SO}}_{2}$, H, and LL as both wavelengths lie on the same side of the isosbestic point at 800 nm. Accordingly, the results for this combination are not shown in Fig. 9.

The estimated H exhibits a linearly decreasing trend, as shown in Figs. 9(c) and 9(g), for PBS and PLS media, respectively, across the same LL range and wavelength combinations. The highest estimation errors in PBS are ∼8.2% and 10.9% for the same wavelength pairs as mentioned earlier, respectively. In PLS medium, the respective errors are 10.2% and 8.4%, respectively. By contrast, Figs. 9(d) and 9(h) reveal that evaluated LL rises linearly with nominal LL for PBS and PLS media, respectively. The estimation error increases as LL grows. In PBS, the average LL estimation errors are ∼13.1% and 19% for the 700-905 and 700-1000 nm combinations, respectively. Similarly, in PLS medium, the same errors are 18.1% and 22.3%, respectively. These values are detailed in Tables 5 and 6, columns 2, 3 and 2, 6; rows 3 to 7.

### 4.2 Experimental Results

Some representative experimentally obtained PA signals at 700, 905, and 1000 nm wavelengths and for LL = 0% and 30% are plotted in Figs. 8(d)–8(f) and 8(j)–8(l) (right block). The PA signal amplitude at LL = 30% is less compared with that of 0%, as also seen in simulations (left block of Fig. 8). Generally, the PA signal strength is higher at 905 nm than at other wavelengths.

In Table 4, pairwise t-test decisions for lysis comparisons, via PA signals, are reported in binary form (0 or 1), based on p-values evaluated at a significance level of α=0.05. At the lysis pairs (0, 6)% and (0, 14)% at 700 nm, PA signal amplitudes are not significantly distinguishable (p<0.05) for donors 1 to 4, irrespective of the sample medium. In contrast, at 905 and 1000 nm, the PA signals do not always remain statistically distinguishable for the (0, 6)% lysis pair. In the remaining cases, statistically significant differences (p≥0.05) are evident in Table 4 as expected. The p-values further decreased with increasing relative difference in LL levels in the pair.

**Table 4:** Binary outcomes of pairwise t-tests derived from calculated p-values comparing experimental PA signal amplitudes between the baseline lysis condition (LL = 0%) and elevated lysis levels (LL = 6% to 30%) at wavelengths of 700, 905, and 1000 nm, measured in PBS and PLS media across all five donors; decision values: 0 = null hypothesis accepted (p-value ≥0.05), 1 = null hypothesis rejected (p-value <0.05).

Donor	Medium	700 nm				905 nm				1000 nm			
		(0,6)	(0,14)	(0,20)	(0,30)	(0,6)	(0,14)	(0,20)	(0,30)	(0,6)	(0,14)	(0,20)	(0,30)
D1	PBS	0	0	1	1	1	1	1	1	0	1	1	1
	PLS	0	0	1	1	0	1	1	1	0	1	1	1
D2	PBS	0	0	1	1	0	0	1	1	1	1	1	1
	PLS	0	0	1	1	1	1	1	1	0	1	1	1
D3	PBS	0	1	1	1	0	1	1	1	1	1	1	1
	PLS	0	0	1	1	0	1	1	1	0	1	1	1
D4	PBS	0	0	1	1	1	1	1	1	1	1	1	1
	PLS	0	0	1	1	0	1	1	1	0	1	1	1
D5	PBS	1	1	1	1	0	1	1	1	1	1	1	1
	PLS	1	1	1	1	1	1	1	1	1	1	1	1

**Fig. 10:**
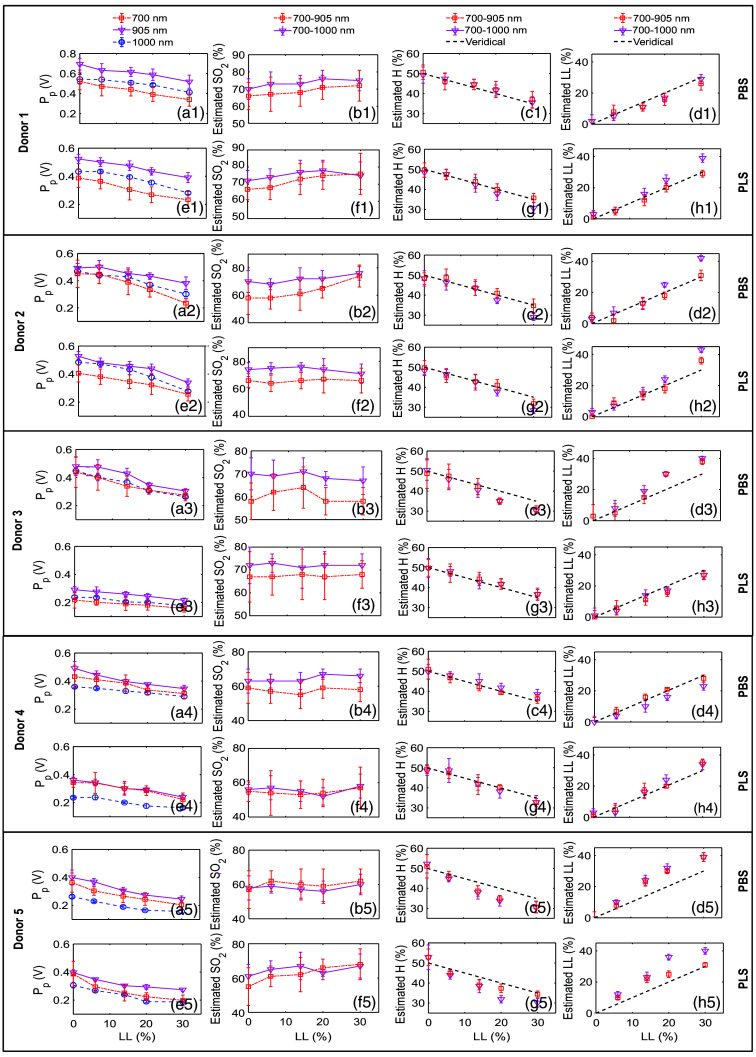
Analysis of experimentally measured PA signals for five different donors (see Table 3 for details) and quantification of the same parameters (mean ± std) using the dual wavelength protocol.

Figure 10, donor 1 to 5, (a1) to (a5), and (e1) to (e5), [column 1, rows 1 to 10] presents the impact of RBC lysis on peak-to-peak amplitude ($P_{p}$, mean ± std) for two different suspending media, at three different incident optical wavelengths and for blood samples collected from five different individuals (elaborated in Table 3). The lysis level is gradually altered from LL = 0 to 30%. The experimental values of $P_{p}$ show a linear decrease with increasing LL. More specifically, ∼35%, 25%, and 24% reductions in PA amplitude can be seen at 700, 905, and 1000 nm wavelengths for donor 1 [see Fig. 10(a1), column 1, row 1].

The ${\mathrm{SO}}_{2}$ levels estimated from experimental data do not exhibit any noticeable variation, though LL has been changed from LL = 0 to 30% as shown in Fig. 10(b1). This trend is followed for both wavelength combinations and in both PBS and PLS suspending media [see Fig. 10, donors 1 to 5, (b1) to (b5), and (f1) to (f5), column 2, rows 1 to 10]. The computed values of blood ${\mathrm{SO}}_{2}$ are also inserted in Tables 5 and 6, columns 5 and 8, rows 8 to 32.

To validate our findings, the same quantity has also been determined from the optical absorbance spectra measured using a UV–Vis spectrophotometer, and the corresponding values are included in columns 4 and 7, Tables 5 and 6. The average ${\mathrm{SO}}_{2}$ estimation errors can be found to be around 14.8%, 11.5% for 700-905 nm and 10.2%, 6.0% for 700-1000 nm wavelength pairs in PBS and PLS media, respectively. The first combination shows slightly better accuracy than the latter wavelength pair.

**Table 5:** Detailed tabulation of the estimated ${\mathrm{SO}}_{2}$ values and lysis levels (LL) determined via numerical and experimental investigations using the wavelength pair of 700–1000 nm.

Blood sample	LL (%)	PBS			PLS		
		Est. LL (%)	Est. ${\mathrm{SO}}_{2}$ (%) UV-Vis	Est. ${\mathrm{SO}}_{2}$ (%) PA	Est. LL (%)	Est. ${\mathrm{SO}}_{2}$ (%) UV-Vis	Est. ${\mathrm{SO}}_{2}$ (%) PA
Simulation	0	0.0	68.0	70.5	–1.0	68.0	70.3
	6	7.0	68.0	70.0	8.0	68.0	70.6
	14	18.0	68.0	69.8	18.0	68.0	69.9
	20	25.0	68.0	69.4	26.0	68.0	70.4
	30	38.0	68.0	68.8	36.0	68.0	70.7
D1	0	2.0 ± 4.0	71.7	70.0 ± 6.0	3.0 ± 2.4	71.7	72.0 ± 6.0
	6	5.0 ± 2.6	—	73.0 ± 7.0	5.0 ± 2.5	—	74.0 ± 5.0
	14	11.0 ± 2.5	—	73.0 ± 5.0	16.0 ± 3.6	—	77.0 ± 7.0
	20	17.0 ± 2.7	—	76.0 ± 5.0	25.0 ± 3.2	—	78.0 ± 6.0
	30	29.0 ± 2.8	74.1	75.0 ± 6.0	39.0 ± 2.7	74.1	75.0 ± 8.0
D2	0	3.0 ± 3.8	72.3	70.0 ± 8.0	3.0 ± 2.4	72.3	74.0 ± 5.0
	6	7.0 ± 3.8	—	68.0 ± 4.0	7.0 ± 2.7	—	75.0 ± 4.0
	14	13.0 ± 3.4	—	72.0 ± 8.0	15.0 ± 3.9	—	76.0 ± 3.0
	20	25.0 ± 1.5	—	72.0 ± 6.0	24.0 ± 2.2	—	74.0 ± 8.0
	30	42.0 ± 1.7	72.2	76.0 ± 5.0	43.0 ± 1.6	72.3	71.0 ± 7.0
D3	0	−1.0 ± 5.0	74.0	70.0 ± 7.0	1.0 ± 4.8	74.0	72.0 ± 8.0
	6	8.0 ± 5.0	—	69.0 ± 7.0	4.0 ± 2.4	—	73.0 ± 4.0
	14	19.0 ± 3.5	—	71.0 ± 6.0	14.0 ± 3.4	—	71.0 ± 9.0
	20	30.0 ± 1.6	—	68.0 ± 3.0	17.0 ± 2.5	—	72.0 ± 6.0
	30	40.0 ± 1.4	73.0	67.0 ± 6.0	27.0 ± 2.3	73.0	72.0 ± 5.0
D4	0	0.0 ± 3.7	73.8	63.0 ± 7.0	3.0 ± 2.4	73.8	56.0 ± 3.0
	6	4.0 ± 1.9	—	63.0 ± 4.0	3.0 ± 5.9	—	57.0 ± 7.0
	14	10.0 ± 3.6	—	63.0 ± 5.0	16.0 ± 3.1	—	55.0 ± 6.0
	20	16.0 ± 2.1	—	67.0 ± 3.0	24.0 ± 3.2	—	52.0 ± 5.0
	30	23.0 ± 2.5	71.7	66.0 ± 4.0	34.0 ± 3.4	71.7	58.0 ± 7.0
D5	0	–4.0 ± 4.5	73.6	58.0 ± 7.0	–6.0 ± 6.1	73.6	61.0 ± 7.0
	6	10.0 ± 1.5	—	59.0 ± 4.0	12.0 ± 1.9	—	65.0 ± 5.0
	14	24.0 ± 3.3	—	57.0 ± 5.0	23.0 ± 3.3	—	67.0 ± 8.0
	20	32.0 ± 2.6	—	56.0 ± 6.0	36.0 ± 1.8	—	63.0 ± 4.0
	30	39.0 ± 2.7	72.1	60.0 ± 6.0	40.0 ± 2.1	72.1	67.0 ± 7.0

**Table 6:** Detailed tabulation of the estimated ${\mathrm{SO}}_{2}$ values and lysis levels (LL) determined via numerical and experimental investigations using the wavelength pair of 700-905 nm.

Blood sample	LL (%)	PBS			PLS		
		Est. LL (%)	Est. ${\mathrm{SO}}_{2}$ (%) UV-Vis	Est. ${\mathrm{SO}}_{2}$ (%) PA	Est. LL (%)	Est. ${\mathrm{SO}}_{2}$ (%) UV-Vis	Est. ${\mathrm{SO}}_{2}$ (%) PA
Simulation	0	0.0	68.0	67.9	0.0	68.0	68.3
	6	7.0	68.0	66.9	8.0	68.0	69.0
	14	16.0	68.0	68.3	16.0	68.0	69.3
	20	23.0	68.0	67.6	24.0	68.0	69.8
	30	36.0	68.0	67.5	37.0	68.0	68.3
D1	0	−1.0 ± 3.6	71.7	66.0 ± 8.0	1.0 ± 3.5	71.7	67.0 ± 6.0
	6	8.0 ± 4.2	—	67.0 ± 10.0	5.0 ± 2.5	—	68.0 ± 7.0
	14	11.0 ± 2.6	—	68.0 ± 8.0	12.0 ± 3.5	—	73.0 ± 9.0
	20	16.0 ± 4.0	—	71.0 ± 7.0	20.0 ± 2.7	—	75.0 ± 8.0
	30	26.0 ± 4.1	74.1	72.0 ± 9.0	29.0 ± 2.1	74.1	76.0 ± 12.0
D2	0	4.0 ± 2.8	72.3	58.0 ± 12.0	0.0 ± 3.5	72.3	66.0 ± 5.0
	6	2.0 ± 4.2	—	58.0 ± 8.0	9.0 ± 3.2	—	64.0 ± 6.0
	14	13.0 ± 3.9	—	61.0 ± 12.0	14.0 ± 3.2	—	66.0 ± 6.0
	20	18.0 ± 2.3	—	65.0 ± 7.0	18.0 ± 2.7	—	67.0 ± 10.0
	30	31.0 ± 3.3	72.2	74.0 ± 8.0	36.0 ± 2.2	72.3	66.0 ± 9.0
D3	0	3.0 ± 7.3	74.0	58.0 ± 8.0	0.0 ± 4.2	74.0	67.0 ± 11.0
	6	5.0 ± 5.9	—	62.0 ± 8.0	6.0 ± 4.6	—	67.0 ± 8.0
	14	15.0 ± 4.0	—	64.0 ± 9.0	11.0 ± 3.3	—	68.0 ± 11.0
	20	30.0 ± 1.4	—	58.0 ± 6.0	16.0 ± 2.7	—	67.0 ± 10.0
	30	38.0 ± 1.9	73.0	58.0 ± 6.0	27.0 ± 3.0	73.0	68.0 ± 6.0
D4	0	—2.0 ± 4.9	73.8	59.0 ± 9.0	1.0 ± 2.2	73.8	55.0 ± 6.0
	6	7.0 ± 2.3	—	57.0 ± 7.0	5.0 ± 2.8	—	54.0 ± 13.0
	14	16.0 ± 1.9	—	55.0 ± 8.0	17.0 ± 4.9	—	53.0 ± 8.0
	20	21.0 ± 0.6	—	59.0 ± 6.0	20.0 ± 1.3	—	54.0 ± 8.0
	30	28.0 ± 2.2	71.7	58.0 ± 7.0	35.0 ± 2.0	71.7	57.0 ± 12.0
D5	0	−2.0 ± 5.8	73.6	57.0 ± 11.0	–6.0 ± 4.0	73.6	55.0 ± 11.0
	6	8.0 ± 2.2	—	62.0 ± 6.0	10.0 ± 1.5	—	61.0 ± 6.0
	14	23.0 ± 2.6	—	60.0 ± 9.0	22.0 ± 2.4	—	62.0 ± 10.0
	20	30.0 ± 1.6	—	59.0 ± 10.0	25.0 ± 2.1	—	66.0 ± 5.0
	30	39.0 ± 2.8	72.1	62.0 ± 7.0	31.0 ± 1.5	72.1	68.0 ± 9.0

Note that the PA method appears to facilitate the physiologically meaningful estimation of the blood ${\mathrm{SO}}_{2}$ level, as venous blood samples were collected from healthy individuals whose ${\mathrm{SO}}_{2}$ levels lie roughly between 60% and 80%. The determined H decreases linearly as LL increases [see Fig. 10, donors 1 to 5, (c1) to (c5) and (g1) to (g5), column 3, rows 1 to 10]. It is clear from these figures that the PA method works very well up to LL≤10%.

However, its deviation from the veridical line becomes pronounced at higher LL values, particularly at LL = 30% for the wavelength combinations 700-905 and 700-1000 nm and for both PBS and PLS media across all five donors.

The estimated LL values also vary linearly with nominal LL levels, as depicted in Fig. 10, donors 1 to 5, (d1) to (d5), and (h1) to (h5) [column 4, rows 1 to 10] and Tables 5 and 6, columns 2, 3, 6 and rows 3 to 32. As expected, the mismatch between the evaluated and actual values is prominent at higher lysis levels, specifically at LL = 30%. In general, the measured data for donor 5 demonstrate greater variability compared with the remaining donors.

## 5 Discussion

One of the primary contributions of this study is the adoption of the biconcave shape of RBCs, as opposed to the commonly assumed spherical geometry. The DDA method was employed to compute the absorption and scattering coefficient factors as well as the anisotropy factor for a single RBC. These parameters were evaluated for various surrounding media (PBS, PLS, PBS/PLS based lysed media). Subsequently, optical parameters, namely, $\mu_{\mathrm{abs}}^{\mathrm{BS}}$, $\mu_{\mathrm{sca}}^{\mathrm{BS}}$, and g, were estimated for different blood samples. The g values provided by the DDSCAT are found to be >0.9 (see Table 2). For RBCs, with a size greater than the illumination wavelength, the scattering is highly forward-directed, leading to a high value of the anisotropy parameter, typically in the range of 0.9 and above. Furthermore, as one would expect, the refractive index of the surrounding medium also increases with increasing percentage of hemolysis. This leads to a better matching of the refractive index between the medium and the scatterer (RBC here), which effectively narrows the forward scattering profile, thereby increasing the anisotropy parameter value even further.

The optical parameters control photon propagation vis-á-vis PA emission from a tissue. DDA provides highly accurate results, but it is computationally intensive and time-consuming. For example, for a single RBC with 125 orientational averages, computation time was ∼3.3  h at 1000 nm, whereas the time requirement was ∼12  h at 450 nm. Therefore, the computational challenge intensifies at lower wavelengths. It is worth noting that the number of dipoles used in this study was $∼16\times 10^{5}$ at 1000 nm and $90\times 10^{5}$ at 450 nm. The DDA method may not be suitable for numerically quantifying the optical parameters of blood samples containing clots or aggregates resulting from multicellular interactions. Therefore, alternative methods are needed to efficiently estimate these parameters in such complex biological systems. MC simulations—especially GPU-accelerated versions such as MCX—are widely used for estimating bulk optical properties in turbid media.^67^ Finite-difference time-domain and finite element method approaches offer full-wave and multiphysics capabilities, making them well suited for complex or heterogeneous tissues.^68^^,^^69^ These alternatives enable scalable and practical optical modeling beyond the scope of DDA.

The numerical values of absorption coefficient, $\mu_{\mathrm{abs}}^{\mathrm{BS}}$, of the blood samples at different lysis levels considered in this study were calculated using Eq. (15) and are tabulated in Table 1, columns 7 and 11, rows 3 to 17. The absorption values remain constant across all lysis levels at each wavelength. This is because light absorption depends on the total hemoglobin concentration, which remains constant with lysis because the initial hematocrit was fixed at H = 50%. The calculated values of $\mu_{\mathrm{abs}}^{\mathrm{BS}}$ provided by the DDSCAT algorithm look very interesting (see Table 2, columns 4 and 9; rows 3 to 17). It decreases with increasing lysis level. This behavior essentially supports the notion that photons may have undergone multiple reflections within erythrocytes (due to the presence of the cell membrane).^70^ Notably, as RBCs experience lysis, the sample became more homogeneous, leading to a reduction in the scattering coefficient ($\mu_{\mathrm{sca}}^{\mathrm{BS}}$) and an expected increase in the anisotropy factor (g). These variations can also be seen from Table 2, columns 6, 7; 11, 12; rows 3 to 17). The changes of $\mu_{\mathrm{abs}}^{\mathrm{BS}}$, $\mu_{\mathrm{sca}}^{\mathrm{BS}}$, and PA amplitude with RBC lysis (columns 1 of Fig. 9 and Fig. 10) are consistent with published results (see Figure 10 of Ref. 70).

It is clear from this study that PA signal amplitude decreases with increasing lysis level, though the total Hb content is the same in each group (say the samples for donor 1) as considered in this study. Thus, the estimated THB or H gradually decreases with increasing lysis (see the third column of Fig. 10). The dual-wavelength PAs thus provide us the THB level that comes from the Hb and HbO enclosed in cells. It excludes the freely suspending Hb and HbO. This is an important finding of this work. As far as we know, it has not been reported previously. This observation helps us to estimate the LL level of a lysed sample if its initial H level is known.

The estimated ${\mathrm{SO}}_{2}$ levels are presented in Tables 5 and 6. In general, it can be seen that the ${\mathrm{SO}}_{2}$ levels in Table 6 have a larger deviation between UV–Vis and PA measurements. This is consistently observed for all donors. The underlying reason behind the observed deviation is that two measurements are performed at highly different concentration levels. Although the UV–Vis measurement requires dilution down to a hematocrit level of 0.04%, the PA measurements are performed at clinically relevant hematocrit levels. The optical absorption contrasts for HbO and Hb are different at 700-1000 and 700-905 nm wavelength pairs. It may be noted that the ${\mathrm{SO}}_{2}$ values for donors 4 and 5 are predicted to be very low. Nevertheless, for donor 4, the H level of the same donor is >55% as given in row 5, column 4 of Table 3. Although no clinical conclusion can be drawn from the present data, the coexistence of an elevated H and comparatively low ${\mathrm{SO}}_{2}$ levels in donor 4 may point to a possible underlying hematological alteration, such as polycythemia.^71^^,^^72^ Such conditions are known to be associated with increased RBC concentration and reduced oxygen carrying capacity; however, this interpretation remains speculative and would require independent clinical validation.

**Fig. 11:**
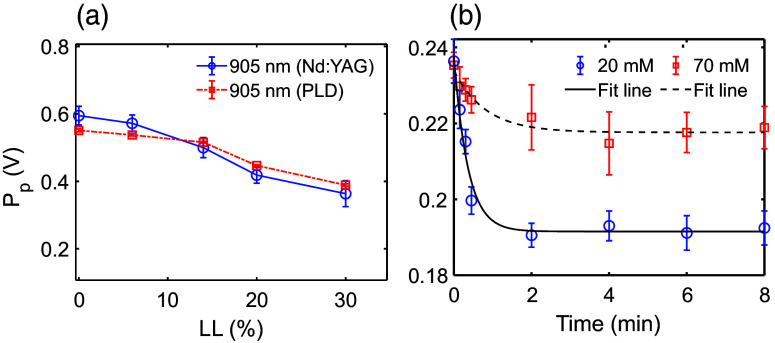
(a) Plots of PA signal amplitude as a function of lysis level (static) for 905 nm optical illumination, measured using both Nd:YAG and PLD sources with PBS as the suspending medium. (b) PA response during the lysis process (dynamic). Packed RBCs with H = 25% have been mixed with saline media with 20 mM and 70 mM NaCl concentrations.

In this study, we performed PA measurements on various lysed blood samples (static). However, the PA technique can also be deployed to monitor the dynamic variation of RBC lysis. To study this aspect, a custom-made PLD device emitting a 905 nm laser beam was employed. A blood sample was collected from a single donor, and the lysis level was varied within the same range, LL = 0–30%. The PA signals obtained from Nd:YAG and PLD systems are compared in Fig. 11(a). As expected, the normalized PA amplitudes measured by both systems were found to be nearly identical. The plots of PA peak-to-peak amplitude as a function of time (lysis process) are illustrated in Fig. 11(b) for two representative blood samples (RBCs are suspended in 20 and 70 mM NaCl concentrated saline water). An increase in salt concentration leads to a delayed lysis of RBCs, attributed to the decrease in osmotic pressure exerted on the cell membranes, which can be clearly seen from Fig. 11(b). Banerjee et al. also observed the same trend.^24^

It is worth noting that RBCs undergo progressive biochemical and structural changes during storage, including ATP depletion, increased membrane rigidity, oxidative stress, and ion imbalance. These changes make them more prone to lysis. Factors such as storage temperature, additive solutions, and leukoreduction have a significant impact on RBC viability. In addition, exposure of phosphatidylserine and hemoglobin release due to lysis can trigger inflammation and transfusion-related complications, emphasizing the need for proper storage and monitoring of blood bags.^73^^,^^74^ The methodology reported herein may be applied to examine the health of stored blood. As found, 700-905 nm or 700-1000 nm wavelength pairs may be utilized for simultaneous quantification of the oxygenation, hematocrit, and lysis levels of such blood samples. Attempts will be made in the future to achieve this end. The technique may be further developed to quantify the lysis levels *in vivo*, which may be useful for monitoring patients. Note that this method can accurately quantify the lysis level when the initial and present hematocrit levels are estimated accurately. In practice, it is unlikely that one has the knowledge of initial H beforehand. This challenge may be solved using machine learning (ML), deep learning (DL), and artificial intelligence (AI) approaches, which may enable accurate and concurrent estimations of H, LL, and ${\mathrm{SO}}_{2}$ *in vivo*. Accordingly, statistical analysis will also be conducted to examine the sensitivity and specificity of the PA method.

## 6 Conclusion

The study investigates the lysis process of RBCs using both numerical and experimental tools involving PAs. The DDA effectively captured the structural effects of RBCs, despite initial refractive index calculations did not incorporate cellular features. The estimation accuracy for H reached up to 90% for 700-905 and 700-1000 nm wavelength pairs up to LL = 14%. Interestingly, ${\mathrm{SO}}_{2}$ remained relatively stable across the LL range of 0% to 30%. The nominal and predicted LL levels are found to be strongly correlated with a correlation coefficient of ≈90%. Among all tested combinations, the wavelength pairs 700-905 and 700-1000 nm have been found to be optimal for the simultaneous determination of H, LL, and ${\mathrm{SO}}_{2}$ parameters of blood samples in practice.

## Data Availability

All data needed to evaluate the conclusions in the paper are present in the paper. Additional data related to this paper may be requested from the authors.
